# Combination of NTP with cetuximab inhibited invasion/migration of cetuximab-resistant OSCC cells: Involvement of NF-κB signaling

**DOI:** 10.1038/srep18208

**Published:** 2015-12-14

**Authors:** Jae Won Chang, Sung Un Kang, Yoo Seob Shin, Seong Jin Seo, Yeon Soo Kim, Sang Sik Yang, Jong-Soo Lee, Eunpyo Moon, Keunho Lee, Chul-Ho Kim

**Affiliations:** 1 Department of Otolaryngology-Head and Neck Surgery, College of Medicine, Chungnam National University, Daejeon, Republic of Korea; 2 Department of Otolaryngology, School of Medicine, Ajou University, Suwon, Republic of Korea; 3 Department of Electrical and Computer Engineering, Ajou University, Suwon, Republic of Korea; 4 Department of Molecular Science and Technology and Department of Life Science, Ajou University, Suwon, Republic of Korea; 5 PSM America Inc., Colorado Springs, CO, USA.

## Abstract

Although the epidermal growth factor receptor (EGFR) is an established target in head-and-neck cancer (HNC), resistance to EGFR-targeted therapy mediated by various mechanisms has been reported. Therefore, a combination strategy to overcome resistance to EGFR mono-targeted therapy is clinically required. We have previously demonstrated that non-thermal atmospheric pressure plasma (NTP) induces death of various cancer cells, including oral squamous cancer (OSCC) cells. In this study, we report for the first time that combining NTP treatment with cetuximab led to inhibition of migration and invasion in cetuximab-resistant OSCC cells, which could be a promising strategy to overcome resistance to anti-EGFR therapy. NTP induced deactivation of NF-κB in SCCQLL1 cells, but not in MSKQLL1 cells. In addition, NTP increased the expression level of E-cadherin, and decreased those of vimentin, Slug, Snail, matrix metalloproteinase (MMP)-2, -9, and activities of MMPs. Moreover, NF-κB upregulation using cDNA diminished the combination effect of NTP on invasion, migration and related signals. Taken together, these results indicate that the combination of NTP with cetuximab can decrease invasiveness in cetuximab-resistant OSCCs through a novel mechanism involving the NF-κB pathway. These findings show the therapeutic potential of treatment that combines NTP and cetuximab in OSCC.

Oral squamous cell carcinoma (OSCC) is one the most frequent head-and-neck cancer (HNC), accounting for ~3% of all newly diagnosed cancer cases^1^. Despite recent advances in surgery, radiotherapy and chemotherapy treatment protocols, the long-term survival of patients with OSCC has remained almost unchanged over the past decade^2^. Therefore, new therapeutic strategies, including molecular-targeted therapies, are needed.

Epidermal growth factor receptor (EGFR) is a well-established molecular target that has been implicated in the pathogenesis and prognosis of OSCC. Despite targeting EGFR using various strategies to abrogate tumor growth in preclinical studies, however, only a subset of patients showed responses to EGFR inhibitors, including cetuximab. Accumulating investigations have elucidated various resistance mechanisms to EGFR inhibitors and encouraged the development of combination strategies that can overcome resistance to EGFR monotherapy.

Since plasma–which is an ionized mixture of gas including ions, electron, free radicals, and photons–can be generated and applied at room temperature by virtue of advances in biophysics and technology, it is being actively investigated and applied in various fields including blood coagulation, wound healing, and tissue and device sterilization. Moreover, we recently revealed that non-thermal atmospheric pressure plasma (NTP) can inhibit the invasive character of cancer cells by decreasing matrix metalloproteinase (MMP)-2/-9 and urokinase-type plasminogen activator (uPA) activities and rearranging the cytoskeleton (related with FAK/Src signals^3^), as well as inducing apoptosis and DNA damage, triggering sub-G_1_ arrest in cancer cells^4^,^5^.

In this study, we evaluated whether combined treatment with NTP and cetuximab is a viable alternative tactic for cetuximab resistant OSCC cells and investigated the molecular anticancer mechanism of NTP in combination with cetuximab in terms of the NF-κB signaling pathway. To the best of our knowledge, this is the first report of combination treatment of NTP for circumventing resistance to molecular-targeted therapy.

## Results

### OSCC cell lines showed resistance to cetuximab monotherapy regardless of EGFR expression

**Figure 1:**
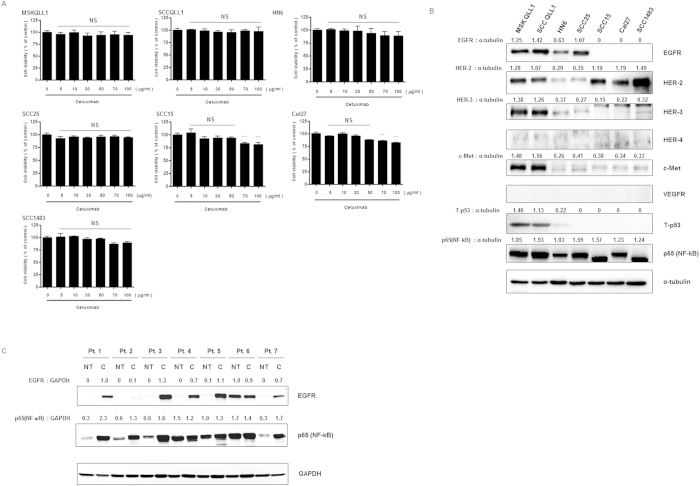
Cetuximab-resistant OSCC cells have increased NF-κB expression regardless of EGFR expression. (**A**) Dose-dependent effect of cetuximab on proliferation of OSCC cell lines. Cells were plated for 24 h, followed by treatment with each concentration of cetuximab for 24 h. Cell viabilities were evaluated by MTT assay. The data represent the means ± S.D. of three independent experiments performed in triplicate. NS, not significant; **P < 0.01. (**B**) Western blot assay of cetuximab-resistant OSCC cell lines for known signals associated with EGFR resistance. (**C**) Western blots of tumor tissue (T) and non-tumor tissue (NT), harvested from patients with cetuximab-resistant OSCC. Pt., patient. Each Western-blotting band was representative of three experiments performed in triplicate.

To determine whether cetuximab, which is a competitive inhibitor of the EGFR pathway and approved for HNC in the clinical setting, has a cytotoxic effect on oral cancer cells, we first performed a proliferation assay. As shown in Fig. 1A, no significant cell death was induced by cetuximab treatment alone in squamous cell carcinoma lines originating from human oral cancer (MSKQLL1, SCCQLL1, HN6, SCC25, SCC15, Cal27, and SCC1483) up to the 50 μg/ml concentration.

Next, we identified the constitutive expression of EGFR (HER-1) and other cell surface receptors or intracellular molecules, which are associated with sensitivity or resistance to EGFR inhibition, such as HER-2, -3, -4, c-Met, VEGFR, p53, and p65 (NF-κB). As shown in Fig. 1B, MSKQLL1, SCCQLL1, HN6, and SCC25 cells showed resistance to EGFR inhibition despite EGFR overexpression. Although MSKQLL1 and SCCQLL1 cells showed overexpression of various surface molecules related to low sensitivity to EGFR inhibition such as HER-2, -3, c-Met, and p53 and these overexpressions of EGFR resistance related signals may explain the reason of nearly complete resistance to cetuximab of both cell lines, all of the oral cancer cells analyzed in this study, interestingly, showed intense expression of NF-κB.

Subsequently, to confirm the above-mentioned results in the human tissue, we evaluated EGFR and NF-κB expression in cetuximab-resistant tumor tissue harvested from oral cancer patients. Among the seven patients, five showed intense EGFR overexpression in cancer tissue compared with normal tissue. One patient had scarce EGFR expression in both normal and cancer tissues, while the other patient showed intense overexpression in both tissues. Consistent with the *in vitro* data, all cancer tissues showed significantly higher expression of NF-κB than did normal tissue (Fig. 1C).

### Combination of NTP with cetuximab had no significant cytotoxic effect in OSCC cell lines

**Figure 2:**
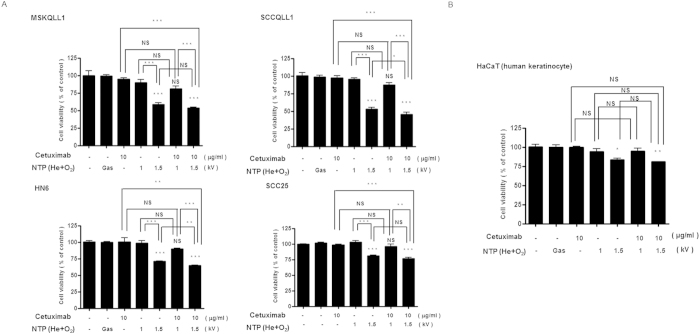
Combination of NTP with cetuximab has no significant cytotoxic effect in EGFR-positive OSCC cell lines. After overnight incubation in 96-well plates, the cells were treated with gas only or with NTP jets at 1 or 1.5 kV for 1 sec with/without 10 μg/ml of cetuximab, and then incubated for 24 h. MTT assay of the viability of (**A**) EGFR-expressing OSCC cell lines (MSKQLL1, SCCQLL1, HN6, and SCC25), and (**B**) normal human keratinocyte, HaCaT, cells as a representative normal oral cavity epithelial cell. The data represent the means ± S.D. of three independent experiments performed in triplicate. NS, not significant; *P < 0.05, **P < 0.01 and ***P < 0.001.

To evaluate the effect of the combination of NTP and cetuximab other than through cell death, we were willing to minimize the NTP or cetuximab-induced cytotoxic effect in each cancer cell line. As presented in Fig. 2A, all OSCC cells showing high-level EGFR expression (MSKQLL1, SCCQLL1, HN6, and SCC25) did not show significant cell death by treatment with 10 μg/ml cetuximab. In addition, because NTP showed a cytotoxic effect on oral cancer cells with 2-kV plasma intensity^4^, we examined the effect of NTP on cell viability using 1 and 1.5 kV of NTP (Fig. 2A). A gas (He+O_2_)-only treatment was used as a control to exclude the effect of gas. Although 1.5 kV of NTP induced significant death of each cell line, 1 kV of NTP only and its combination with cetuximab (10μg/ml) had no cytotoxic effect on all constitutively EGFR-expressing cell types (Figs 1B and 2A).

Next, to examine the cytotoxicity of the determined NTP intensity and cetuximab dose in normal cells, human keratinocyte HaCaT cells were treated with NTP and/or cetuximab and their viability was analyzed. As shown in Fig. 2B, 1 kV of NTP and/or 10 μg/ml of cetuximab exerted no cytotoxic effect on HaCaT cells.

### Combination of NTP with cetuximab significantly inhibits migration/invasion by OSCC cell lines

**Figure 3:**
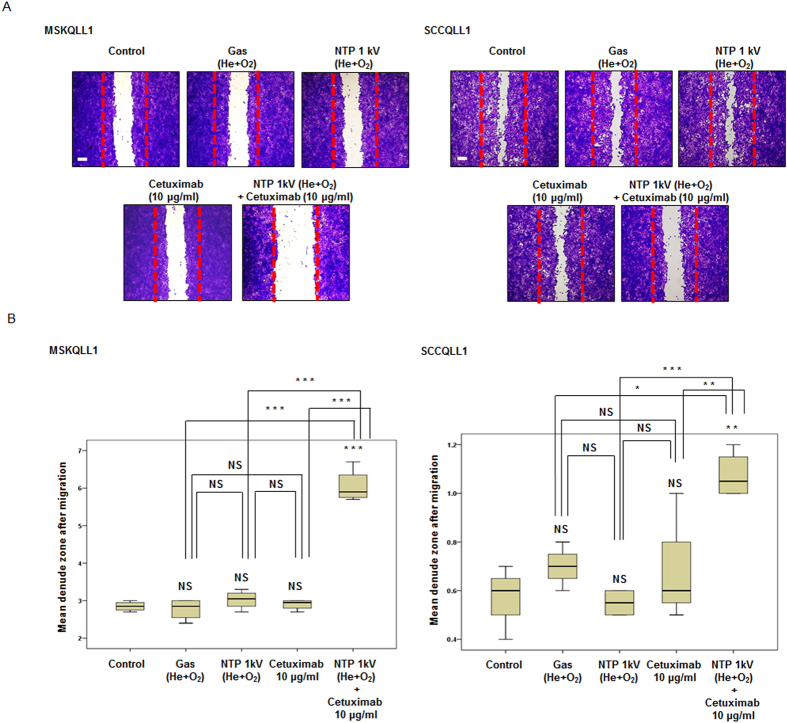
Combination of NTP with cetuximab decreased the migration of MSKQLL1 and SCCQLL1 cells. (**A**) Confluent monolayers of each cell line were wounded by scratching the surface as uniformly as possible using a 1-ml pipette tip. Cells were exposed to gas (He plus O_2_ only), NTP (1 kV) and/or cetuximab (10 μg/ml). After 12 h of incubation, wound healing was evaluated by photography with crystal violet staining. Each figure is representative of three experiments performed in triplicate. Scale bar = 500 μm. (**B**) Mean denuded zone was measured. The data represent the means ± S.D. of three independent experiments. NS, not significant; *P < 0.05, **P < 0.01 and ***P < 0.001.

To investigate whether the combination of NTP and cetuximab treatment reduces tumor cell migration, scratch wound healing assays were performed. As presented in Fig. 3A,B, although treatment with cetuximab (10 μg/ml) or NTP (1 kV) alone did not affect the migration of either cell line, simultaneous treatment with NTP (1 kV) and cetuximab (10 μg/ml) considerably suppressed the migration of MSKQLL1 (*P* < 0.001, *P* < 0.001, *P* < 0.001) and SCCQLL1 (*P* < 0.01, *P* < 0.001, *P* < 0.01) cells across the denuded area compared with control, NTP or cetuximab only, respectively. The percent inhibition of cellular migration in combination group compared with the control was 64.2% in MSKQLL1 cells and 35.6% in SCCQLL1 cells after 12 h of incubation compared to control cells.

**Figure 4:**
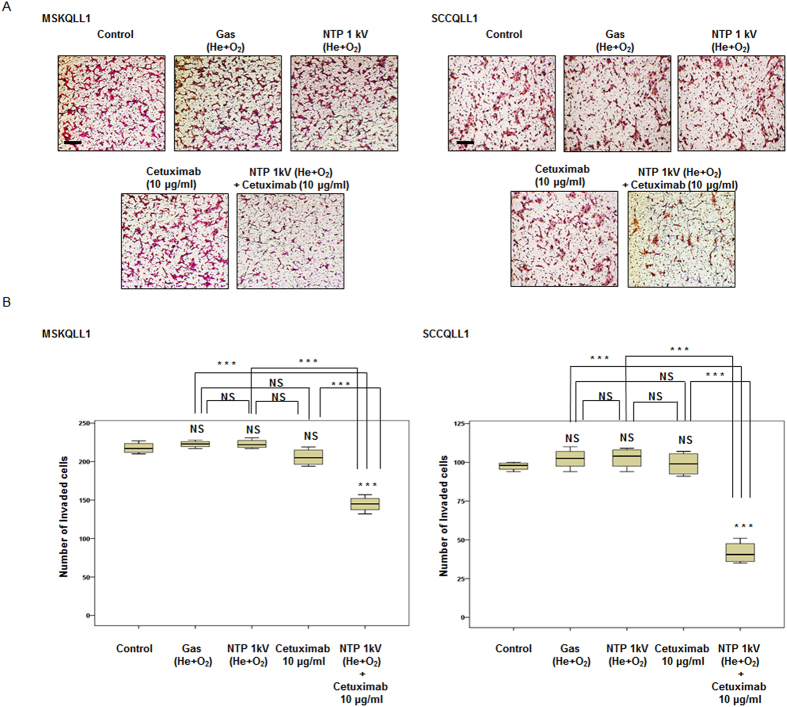
Combination of NTP with cetuximab decreased invasion by both MSKQLL1 and SCCQLL1 cells. (**A**) Each cell line was seeded on a filter (pore size, 8 μm) coated with type I collagen in the upper chamber and exposed to gas (He plus O_2_ only), NTP (1 kV) and/or cetuximab (10 μg/ml). After 24 h, the cells attached to the lower section were stained with H&E. Each figure is representative of three experiments performed in triplicate. Scale bar = 50 μm. (**B**) To quantify invasion, stained cells in the lower chamber were counted using light microscopy (200×). The data represent the means ± S.D. of three independent experiments. NS, not significant; ***P < 0.001.

In addition, to elucidate whether the combination of NTP with cetuximab reduces tumor invasion, type I collagen coated Transwell invasion assays were performed using Boyden chambers. Co-treatment with NTP (1 kV) and cetuximab (10 μg/ml) markedly decreased the number of cells of both cell lines that passed through the filter of the chamber compared with the control, gas only, NTP or cetuximab only, respectively (*P* < 0.001, *P* < 0.001, *P* < 0.001, and *P* < 0.001 for both cell lines), whereas each mono-therapy had no significant effect on invasion capacity. These findings indicate that the combination treatment synergistically inhibited the invasive character of the cancer cell lines evaluated (Fig. 4A,B).

### Combination of NTP with cetuximab regulates the protein expression of NF-κB, p53 and EMT markers in SCCQLL1, but not MSKQLL1, cells

**Figure 5:**
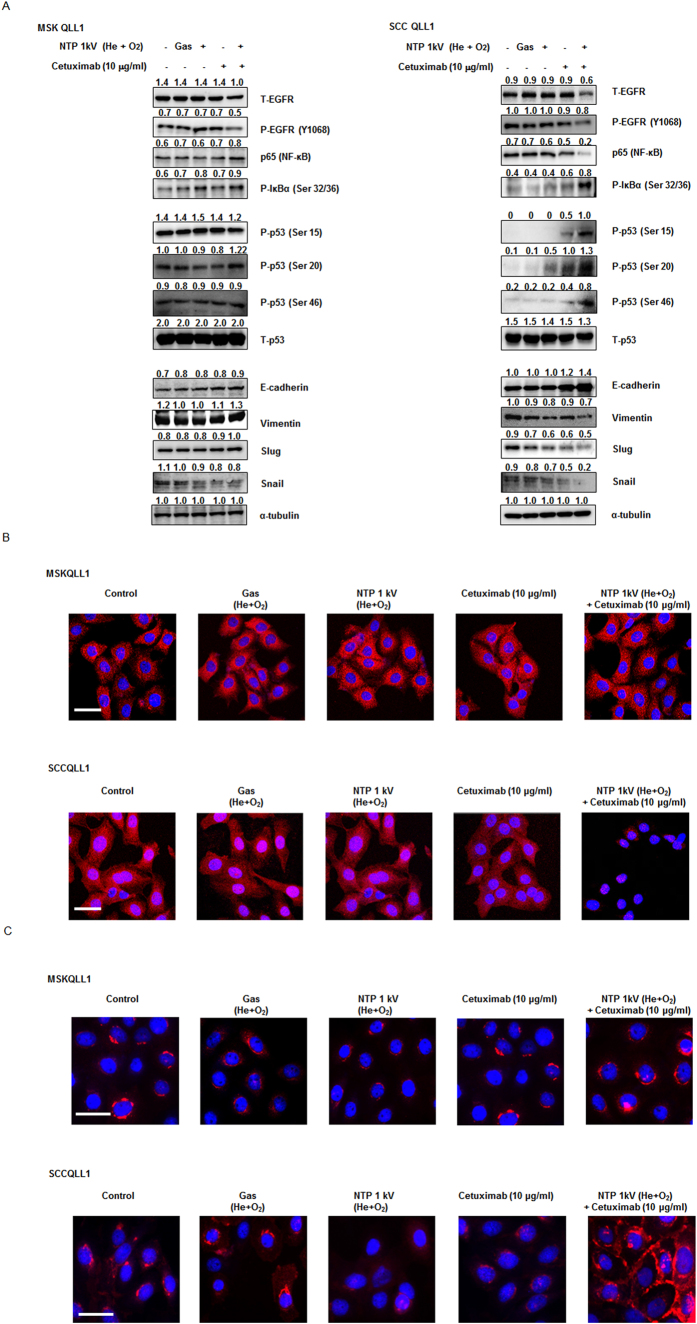
Combination of NTP with cetuximab regulates the protein levels of NF-κB, p53 and EMT markers in SCCQLL1, but not MSKQLL1, cells. (**A**) Western blotting for p-EGFR, p65 (NF-κB), p-IκB, p53, E-cadherin, vimentin, Slug and Snail. Immunocytochemical assay for (**B**) p65 (NF-κB) and (**C**) E-cadherin. Each Western-blotting band is representative of three experiments performed in triplicate. Scale bar = 50 μm.

The transcription factors NF-κB and p53 are critical proteins that are dysregulated in various human cancers, including HNC. In addition, Christine *et al*. reported that epithelial-to-mesenchymal transition (EMT), cell adhesion, and NF-κB pathways are the most prominent molecular characteristics of high-risk HNC using DNA microarrays^6^. Thus, we explored the expression of phospho (P)-EGFR (Y1068), p65 (NF-κB), P-IκBα (Ser 32/36), P-p53 (Ser 15, 20, and 46), E-cadherin, vimentin, Slug, Snail, and MMP-2/-9. As shown in Fig. 5A, the phosphorylation levels of EGFR and NF-κB were significantly decreased in the NTP (1 kV) combination with cetuximab (10 μg/ml) treatment group compared with those in the control, gas, NTP or cetuximab-only groups in SCCQLL1 cells, but not in MSKQLL1 cells. The augmented phosphorylation of IκBα at Ser 32 and Ser 36 residues, which are essential for the control of IκBα stability and the activation of NF-κB^7^, was noted in SCCQLL cells, but not in MSKQLL1 cells. In addition, the expressions of vimentin, Slug and Snail were attenuated and that of E-cadherin was augmented by co-treatment with NTP (1 kV) and cetuximab (10 μg/ml), respectively in SCCQLL1, but not MSKQLL1, cells.

To confirm our findings regarding NF-κB and E-cadherin expression, the intracellular localization of each molecule was analyzed using immunocytochemistry. Although gas, NTP, or cetuximab only treatment did not significantly affect NF-κB expression, which was homogenously intense throughout the cytosol, in the combination group, little NF-κB was detected in SCCQLL1 cells, and was absent in MSKQLL1 cells (Fig. 5B). As presented in Fig. 5C, the expression of E-cadherin, which is normally observed at the cell membrane, was augmented after NTP with cetuximab combination therapy in SCCQLL1, but not MSKQLL1, cells.

### Combination of NTP with cetuximab decreased MMP-2/-9 and uPA activity in SCCQLL1 cells, not in MSKQLL1

**Figure 6:**
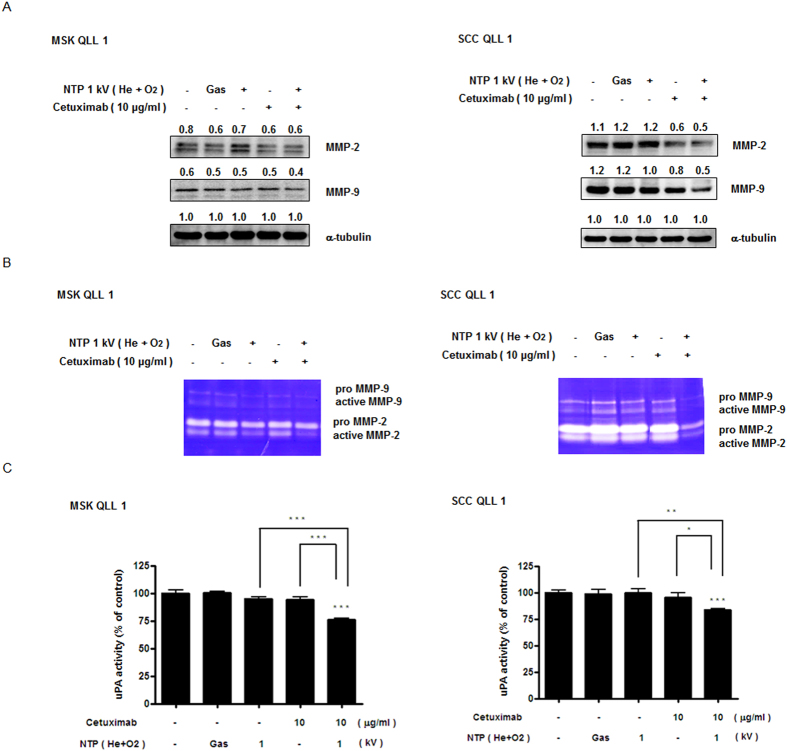
Combination of NTP with cetuximab decreases MMP-2/-9 and uPA activity in SCCQLL1, but not MSKQLL1, cells. After treatment with each condition (gas, 1 kV of NTP, 10 μg/ml of cetuximab, and combination of NTP and cetuximab), (**A**) Western blot analysis for MMP-2/-9 was performed to determine protein levels and (**B**) gelatin zymography for MMP-2/-9 was conducted to evaluate enzyme activity. (**C**) uPA assays. The data represent the means ± S.D. of three independent experiments. NS, not significant; *P<0.05, **P < 0.01 and ***P < 0.001.

MMP-2/-9 and uPA are well-documented pathways related to tumor invasion or metastasis downstream of NF-κB or p53. Thus, to confirm the mechanism by which NTP in combination with cetuximab impacted invasiveness *in vitro*, Western blotting to evaluate the expression of, and gelatin zymography the activity of, MMP-2/-9 were performed. The activity and expression of MMP-2/-9 were reduced by treatment with a combination of NTP (1 kV) plus cetuximab (10 μg/ml) in SCCQLL1, but not MSKQLL1, cells (Fig. 6A,B). The combination treatment also inhibited uPA activity compared with the control and each monotherapy group, as demonstrated by uPA assays (Fig. 6C).

### Combination of NTP with cetuximab attenuates invasion via the NF-κB signaling pathway in SCCQLL1 cells

**Figure 7:**
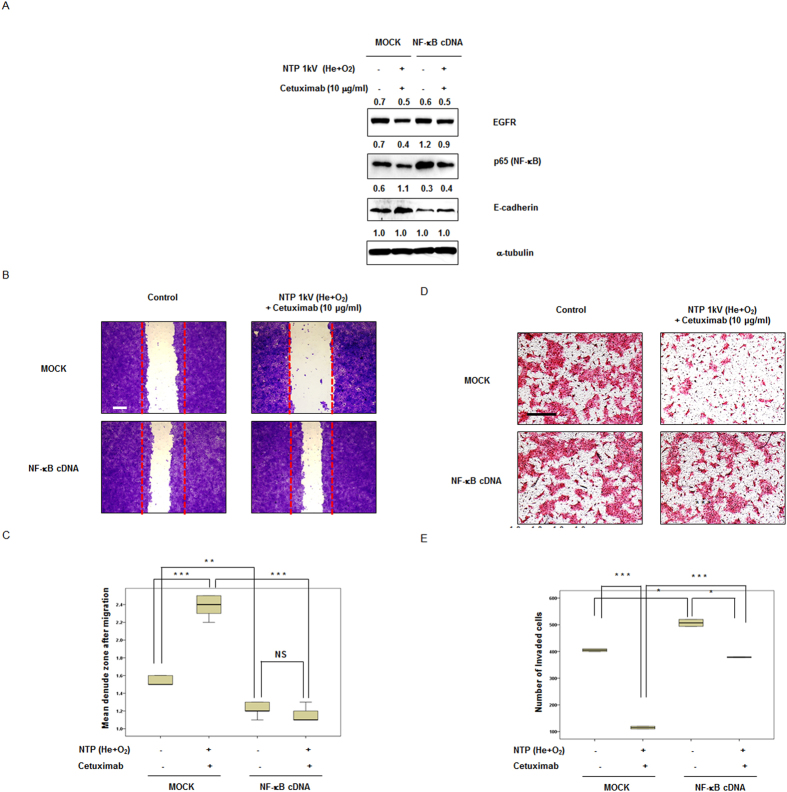
Upregulation of NF-κB attenuated the combination effect of NTP with cetuximab regarding invasive characteristics (migration and invasion) in SCCQLL1 cells. SCCQLL1 cells were flourished NF-κB by cDNA transfection for 24 h, followed by exposure to each condition (gas, 1 kV of NTP, 10 μg/ml of cetuximab, and combination of NTP and cetuximab). (**A**) Western blotting. p65 (NF-κB) protein expression was significantly increased by cDNA transfection; combination treatment of NTP with cetuximab decreased the level of p65 (NF-κB). (**B,C**) Scratch-based migration assay using NF-κB–upregulated SCCQLL1 cells. (**B**) After 12 h of incubation, wound healing was evaluated by photography with crystal violet staining. Each figure is representative of three experiments performed in triplicate. Scale bar = 500 μm. (**C**) Percentage of closure of the denuded zone was analyzed. The data represent the means ± S.D. of three independent experiments. NS, not significant; **P < 0.01; ***P < 0.001. (**D,E**) Invasion assay using NF-κB–upregulated SCCQLL1 cells. (**D**) After exposure to gas (He plus O_2_ only), NTP (1 kV) or/and cetuximab (10 μg/ml), cells were incubated in the upper part of the Transwell chamber for 24 h. Attached cells in the lower section (invading cells) were stained with H&E. Scale bar = 100 μm. Each figure is representative of three experiments performed in triplicate. (**E**) Stained cells in the lower chamber were counted using light microscopy (200×). The data represent the means ± S.D. of three independent experiments. *P < 0.05; ***P < 0.001.

To identify the underlying mechanism of the effect of NTP in combination with cetuximab via the NF-κB pathway, we assessed whether overexpression of NF-κB expression modulated the effect of the combination of NTP with cetuximab. As demonstrated in Fig. 7A, transfection of NF-κB cDNA upregulated NF-κB expression compared with control-transfected cells. As expected, NF-κB augmentation itself noticeably increased migration and invasion of SCCQLL1 cells, and the combination treatment showed no definite significant effect on migration (Fig. 7B,C) and invasion (Fig. 7D,E) of NF-κB upregulated SCCQLL1 cells, respectively.

## Discussion

EGFR is commonly overexpressed or constitutively activated in HNC, including OSCC, and is known to contribute to their uncontrolled proliferation, poor prognosis, and survival^8^. Thus, blocking the EGFR pathway has been regarded as a promising molecular target for HNC^9^. Unfortunately, only 10–20% of patients with HNC tumors display a favorable response to cetuximab monotherapy and even the combination of standard chemotherapies with cetuximab treatment prolongs overall survival by a few months because of resistance to EGFR pathway inhibition^9^,^10^. Therefore, new therapeutic approaches, including rational combination strategies, are needed to increase the long-term survival of OSCC patients.

Evidences from recent literatures suggested NTP as a promising anti-cancer therapeutic method by inducing growth arrest and cell death in various types of cancer cells^3^,^4^,^5^,^11^,^12^,^13^. Although the mechanisms underlying the anticancer effects of NTP have not been fully elucidated, the biological effects of NTP are known to depend mainly on reactive oxygen/nitrogen species (ROS/RNS), which are generated when cells and fluid are brought into contact with NTP^5^,^12^. Previously, we demonstrated that NTP induced anticancer effects via ROS generation^14^. However, we also suggested a novel NTP anticancer mechanism other than ROS signaling^3^,^4^. Especially in thyroid papillary cancer cells, NTP ameliorated the invasive characteristics of cancer cells via FAK inhibition, which is associated with both cytoskeleton modulation and inhibition of MMPs/uPA system activities^3^. The purpose of the current study was to explore the effect of combination treatment with NTP on migration and invasion, rather than cell death, of cetuximab-resistant OSCC cell lines. Thus, all experiments were conducted at a concentration (10 μg/ml cetuximab) or intensity (1 kV NTP) that does not result in cell death even in combination. Moreover, in this study, each mono- or combination therapy alone had no significant effect on HaCaT normal oral epithelial cells.

Our current data indicate that NTP in combination with cetuximab had synergistic antitumor effects by inhibiting migration and invasion of cetuximab-resistant cells via suppression of NF-κB signaling, even though was no significant effect on cancer cell viability. This is the first report to present an anticancer effect of NTP other than apoptosis in OSCC, along with the molecular mechanism, although Guerrero *et al*. suggested the preliminary possibility of non-apoptotic mechanisms without specific mechanistic explanation in human papilloma virus-negative HNC with a minimal effect on the normal adjacent tissue^13^. Furthermore, this is the first report of a strategy comprising the combination of NTP with another anticancer agent in cancer cells to alleviate invasive characteristics (migration/invasion) which are closely associated with locally advancement of tumor or distant metastasis.In normal cells, quiescent NF-κB is activated by inflammatory stimuli. In most cancers, including HNC, NF-κB is involved in tumorigenesis, tumor maintenance or progression, and resistance to cytotoxic chemotherapy^15^. In addition, the abnormal constitutive activation of NF-κB contributes to malignant progression and resistance to therapy^16^. In our data, all cetuximab-resistant cell lines and tumor tissues showed significant NF-κB protein overexpression. Moreover, cetuximab sensitive cells demonstrated few NF-κB expression and down regulation of NF-κB using RNA interference recovered cetuximab sensitivity on cetuximab-resistance cells (Supplementary Figure S1). Thus we postulated that constitutive NF-κB signal activation was related to cetuximab resistance.

Previous studies indicated that NF-κB can induce EMT via various molecular pathways, which differ according to cell type, but resulting in tumor progression and metastasis^17^,^18^ and thus, the NF-κB signal is a potential target for anti-metastatic therapy^17^,^19^. To verify the effect of the combination of NTP with cetuximab on invasive cellular phenotypes at the molecular level, we analyzed protein levels of Slug and Snail, which are transcription factors and master regulators of the epithelial-to-mesenchymal transition (EMT); as well as E-cadherin or vimentin, which are cellular machinery associated with the invasive phenotype of cancer cells and thus hallmarks of the EMT^20^,^21^. Different from our previous report of anti-EMT effects of 2 and 4 kV of NTP^3^, 1 kV of NTP monotherapy did not decrease EMT marker expression and did not inhibit EMT phenotypes, likely because of the low intensity of NTP. However, the combination of NTP with cetuximab showed significant inhibition of both EMT marker expression and characteristics, indicating that the combination treatment exerted a synergistic effect. In addition, because the MMP/uPA system plays an important role in ECM degradation and facilitates tumor migration and invasion^3^,^20^, we assessed the effect of the combination of NTP with cetuximab on the MMP/uPA system. Consistent with the previous report of a relationship between NF-κB and the MMP/uPA system^22^,^23^, we found decreased activities of MMP-2/-9 and uPA as well as decreased MMP-2/-9 protein levels following NTP combination induced NF-κB suppression.

Intriguingly, although a synergistic effect of NTP in combination with cetuximab on the MSKQLL1 and SCCQLL1 cell lines was noted, NF-κB suppression was noted only in SCCQLL1, not MSKQLL1, cells in the present study (among EGFR overexpressing cell lines, NTP combination with cetuixmab also attenuated NF-κB in HN6 cells whereas not in SCC25 cells, Supplementary Figure S2). Because differences in gene expression profiles are related to heterogeneous biological responses, such as sensitivity to chemotherapy and the malignant phenotype of cancer cells^24^, we focused on the differential expression of p53 in the two cell lines. The tumor suppressor protein p53 regulates the cellular response to DNA damage by mediating cell cycle arrest, DNA repair, and cell death^25^. Inactivation of p53 is reported in most human cancers, with mutations in p53 occurring in about 50% of all tumors^24^. In addition to “loss of function” mutants that lack the tumor-suppressive function, “gain of function” p53 mutant variants lose their sequence-specific DNA binding but exert complex DNA interactions instead, thereby modifying the set of target genes related to tumorigenesis and drug resistance^26^. For HNC, about 50% tumors harboring p53 dysfunction related to aberrant overexpression for p53 and inactivation of p53 is observed in most of the rest^24^. Moreover, both the mutation and loss of expression of p53 are implicated in reduced tumor cell apoptosis and resistance to chemotherapy^27^. In our study, similarly, cetuximab resistant cell lines showed aberrant overexpression for p53 or inactivation of p53 with little expression: SCCQLL1 cells showed depleted p53 expression, which was restored by treatment with NTP in combination with cetuximab. On the other hand, MSKQLL1 cells with aberrant overexpression of mutated p53 showed no significant changes in expression regardless of application of mono- or combination therapy. In contrast with SCCQLL1 cells, in MSKQLL1 cells, other mechanisms such as the HER-3 pathway, which is a salvage signal against EGFR inhibition^28^, appear to be linked to the effect of treatment with the combination of NTP with cetuximab (Supplementary Figure S3).

Recent studies demonstrated that besides the constitutive activation of NF-κB, inactivation of p53 is important for invasiveness of many types of cancer^24^,^27^,^29^. p53 has been implicated in malignant progression, metastasis, resistance to chemoradiotherapy, and poorer prognosis through modulating NF-κB activity in several studies^24^,^27^,^30^. Bradford *et al*. demonstrated that wild-type p53 with low expression is related to cisplatin resistance *in vitro*^30^, and chemoradiation resistance in the clinical setting^31^. Tergaonkar *et al*. suggested that p53 stabilization is decreased upon NF-κB activation and that NF-κB is associated with acquisition of chemotherapy resistance^32^. Similarly, Gurova *et al*. reported that decreased expression of p53 can result from NF-κB activation^33^. Furthermore, Tin *et al*. found evidence for an inverse association between inactivation of p53 and NF-κB activation via bioinformatics analysis of genome-wide gene expression data in HNC^24^. These observations support our hypothesis that differential molecular responses (NF-κB suppression or not) to NTP in a subset of OSCC cells (SCCQLL1 and MSKQLL1) is linked to p53 status. In the future, however, it will be necessary to evaluate whether p53 activation is the primary event regulating NF-κB inactivation and the link between p53 activation and NF-κB suppression in OSCC.

Our findings provide new insight into the mechanisms of not only resistance toward cetuximab in OSCC but also treatment with NTP in combination with cetuximab. Several p53-inducing anticancer drugs have been reported to induce p53 as well as NF-κB in various cell types^27^. Under this condition, despite p53-induced apoptosis, NF-κB activation aggravate invasiveness or promote resistance to apoptosis. Therefore, NTP, which has the ability to repress NF-κB signaling associated with cetuximab resistance while also activating the p53 pathway, might possess greater anticancer efficacy. Thus, we can highlight the development of a rational therapeutic strategy comprising NTP in combination with conventional anticancer modalities.

Moreover, we previously presented, the notion of NTP as novel agent for cancer therapy, particularly in oral cavity cancer combined with surgical treatment, because plasma can readily access the site of involvement after primary surgical treatment^4^. In this study, we can advanced our concept of NTP as a novel adjuvant tactic to the locally advanced disease setting.

Locally advanced oral cavity tumors present a significant threat to survival and function and treatment include primary surgical resection with adjuvant chemoradiation therapy, or chemoradiation therapy with surgery reserved for salvage^34^.

When we consider surgical resection of oral cavity cancer primarily, resection margin is a well-known prognostic indicator and it is important to obtain tumor-free resection margins in patients with oral cancer. To achieve this, the surgeon usually removes the tumor with a margin of 10 mm of macroscopically normal tissue, owing to microscopic involvement, and, in more than half of patients with OSCC, the surgical defect is large enough to induce the functional and cosmetic deficit. However, getting the enough surgical margin sometimes seem to be impossible in the locally advanced setting owing to anatomic limitation of oral cavity^4^,^35^. In this case, to apply NTP on the resection bed with postoperative chemoradiotherapy including cetuximab is expected to have synergic anticancer effect and address the tumor-microinvolvement resulting in prevention of local recurrence.

In the primary chemoradiation therapy protocol, combining NTP application on the primary tumor with chemotherapy can be promising tactic of increasing anticancer effect whereas decreasing the toxicity by reducing the dose of chemotherapeutics because of its synergistic anticancer effect. Especially, NTP combination with cetuximab is expected to lowering the delayed treatment failure which is developed by a few portion of cetuximab resistant cells in the whole tumor mass^34^.

Therefore, NTP combination with chemotherapy in OSCC is a more feasible and promising therapeutic strategy than in any other cancer type, in particular to control locoregional failure with selective antitumor capability sparing innocent surrounding tissue, because of the anatomically easy accessibility of this type of tumor and synergic effect.

## Conclusion

We demonstrated that the combination of NTP with cetuximab inhibited invasion/migration of OSCC cells by simultaneously modulating the p53 and NF-κB signaling pathways. Although further investigation, including clinical trials, is needed to prove the usefulness of NTP in combination with cetuximab, it may be a useful and novel strategy for OSCC treatment.

## Materials and Methods

### Cell lines and reagents

Among seven squamous cell carcinoma lines originating from human oral cancer (MSKQLL1, SCCQLL1, HN6, SCC25, SCC15, Cal27, and SCC1483), MSK QLL1 and SCC QLL1 cells were kindly provided by Prof. Se-Heon Kim (Yonsei University, Korea). The others were purchased from the American Type Culture Collection (ATCC, Manassas, VA, USA). HaCaT cells, derived from human keratinocytes, were obtained from the ATCC. MSKQLL1, SCC25, and SCC15 cells were maintained in Dulbecco’s Modified Eagle’s Medium: Nutrient Mixture F-12 (DMEM/F12; GIBCO, Carlsbad, CA, USA). SCCQLL1, HN6 and SCC1483 cells were cultured in Minimum Essential Medium (MEM; GIBCO, Carlsbad, CA, USA). Cal27 and HaCaT cells were cultured in high- and low-glucose Dulbecco’s Modified Eagle’s Medium (DMEM; GIBCO, Grand Island, NY, USA), respectively. All growth media were supplemented with 10% fetal bovine serum (FBS) and penicillin-streptomycin at 100 U/mL (GIBCO) at 37°C with 5% CO_2_ under humidified conditions.

### Tumor tissue samples from patients with OSCC

Tumor biopsies were obtained from primary lesion of randomly selected 7 patients in the cohort of patients with cetuximab resistant OSCC at Ajou University Medical Center. In all patients, cetuximab was treated to each patient at the initial dose of 400 mg/m^2^, followed by weekly infusion of 250 mg/m^2^ and disease assessment was performed every 2 months according to the RECIST criteria^36^. The study was approved by the Institutional review board of Ajou University School of Medicine and was conducted in accordance with ethical principles stated in the most recent version of the Declaration of Helsinki.

### Experimental system specifications & NTP treatment

We developed and produced a spray-type NTP system with a newly designed arc-free and antistatic plate to generate a homogenous cold (non-thermal) plasma jet for biomedical research applications, as described previously^3^,^4^. The plasma jet-generating system was certified to be safe for use for surface modification of biomaterials at a low temperature, which is fundamental for biological experiments^37^.

Specifications of the power supply were as follows: 1-kV minimum, 13-kV maximum, and mean AC voltage frequency of ~15–30 kHz; these specifications were altered markedly according to the type and quantity of gas used. In this study, helium (He) and oxygen (O_2_) were used as carrier gases (10 L/min flow rate) based on our previous study, which revealed that the addition of oxygen to helium plasma resulted in optimum cancer cell deterioration^38^.

For NTP treatment, we used 3 ml of cell suspension with a concentration of 1 × 10^5^ cells/ml on a petri dish (diameter ~60 mm, 10060, SPL, Pochen-Si, Gyeonggi-do, Korea). The distance between the plasma hand-held device and the bottom of the petri dish was maintained at ~3 cm^5^.

### Cell proliferation assay

After seeding the cells in 96-well plates at a density of 5 × 10^3^ cells/well, the effect of cetuximab or/and NTP treatment on cell viability was analyzed 24 h after treatment using an assay based on the conversion of 3-(4,5-dimethylthiazol-2-yl)-2,5-diphenyl-tetrazolium bromide (MTT; Sigma Aldrich) as described previously^39^. Briefly, after addition of MTT solution to the cell suspension (40 μl) for 4 h, the remnant formazan product was dissolved in 100 μl of DMSO. The optical density of each well was measured using a microplate reader (Bio-Tek, Winooski, VT, USA) at 540 nm. The results are presented as percentages relative to control cells.

### Wound-healing assays

For the cell migration assays, cells were plated in 12-well culture plates at a density of approximately 5 × 10^4^/well and grown to confluency. Wound-healing assays were performed as described previously^3^. In brief, the monolayer was scratched with a sterile pipette tip, followed by extensive washing to remove cellular debris. The cells were then exposed to gas (He plus O_2_ only), 1 kV of NTP or/and 10 μg/ml of cetuximab, for 1 s. Wound-healing ratios were documented by photography after 12 h as the doubling times for MSKQLL1 and SCCQLL1 were 13.0 ± 0.3 h and 16.4 ± 1.3 h, respectively. The doubling times were calculated from the cell growth curve over three days, as follows: 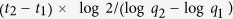 (t_2_: final time, t_1_: initial time, q_2_: final cell number, q_1_: initial cell number).

### Invasion (Transwell) assays

The invasion ability of each cancer cell line was evaluated using Transwell (24-well) chambers (Costar, Cambridge, MA, USA), as described previously^3^. Initially, type I collagen (8 μg/filter) was dissolved in 100 μl of MEM and poured into the upper part of the polyethylene filter (pore size, 8 μm). The wells were coated overnight in a laminar flow hood. Then, 1 × 10^4^ cells (in 100 μl of growth medium) were added to the top of the filter in the upper well. The chamber was incubated for 24 h in 5% CO_2_ at 37°C. Finally, attached cells in the lower section (invading cells) were stained with H&E and counted in four representative fields by light microscopy (×200 magnification).

### Western blot analyses

Cells were lysed in RIPA buffer (Thermo Fisher Scientific, Rochester, NT, USA) containing 25 mM Tris-HCl (pH 7.6), 150 mM NaCl, 1.0% nonidet-P 40 (NP40), 1.0% sodium deoxycholate, 0.1% sodium dodecyl sulfate (SDS) with protease inhibitor cocktail and PhosphoSTOP (Roche Applied Science, Vienna, Austria, pH 7.4), and prepared as described previously^4^. The following antibodies were used for Western blotting analysis: anti-phospho-p65 (NF-κB), -phospho-IκBα, -phospho-p53 (Ser 15, 20, and 46), -phospho-EGFR, -E-cadherin, -vimentin, -Slug, -Snail, -MMP2/9, and α-tubulin (Cell Signaling Technology, Danvers, MA, 1:1000).

### Zymography

MMP-2/-9 activity was assayed using gelatin zymography as described previously^3^. MSK QLL1 and SCC QLL1 cells were treated with gas (He+O_2_) only, 1 kV of NTP for 1 s, 10 μg/ml of cetuximab, and NTP (1 kV) plus cetuximab (10 μg/ml), and incubated for an additional 24 h. The supernatant (100 μl) from each sample was mixed with 1 μl of 100 mM 4-aminophenylmercuric acetate, and the samples were activated for 1 h at 37°C. Next, each sample was placed in sample buffer for 10 min and electrophoresed in polyacrylamide gels at 125 V for 120 min at 4°C using a Novex Xcell II system (Life Technologies, Carlsbad, CA, USA). The gels were incubated in renaturation buffer for 60 min at room temperature, followed by incubation for 18 h in 100 ml of developing buffer at 37°C with light shaking. The gels were then stained for 3 h with Coomassie brilliant blue. After decolorization in 400 ml of methanol, 100 ml of acetic acid, and 500 ml of distilled water, images were obtained using an image analyzer.

### Urokinase-type plasminogen activator (uPA) assays

MSKQLL1 and SCCQLL1 cells (3000 cells/well) were added to 96-well plates in complete medium containing 10% FBS. After overnight incubation, the cells were treated with gas (He+O_2_) only, 1 kV of NTP, 10 μg/ml of cetuximab, and NTP (1 kV for 1 s) plus cetuximab (10 μg/ml), respectively. The plates were then incubated for a further 24 h. The cells were then processed as described previously. Briefly, cells were washed with DMEM lacking phenol red and placed in 200 μl of reaction buffer containing 50% (v/v) of 0.05 U/ml plasminogen in DMEM (without phenol red), 40% (v/v) of 50 mM Tris-buffer (pH 8.2), and 10% (v/v) of 2.25 mM chromozyme PL in 100 mM glycine. The mixtures were incubated for 3 h at 37°C in 5% CO_2_. The absorbance at 405 nm was measured using an automated spectrophotometric plate reader.

### Immunocytochemistry

After culture on a microscope cover glass (Thermo Fisher Scientific, Rochester, NY, USA), cells were treated with either gas (He+O_2_) only, 1 kV of NTP, 10 μg/ml of cetuximab, and NTP (1 kV for 1 s) plus cetuximab (10 μg/ml). After a 24-h incubation, cells were fixed with 4% formaldehyde and blocked in bovine serum albumin (BSA) in 5% phosphate-buffered saline (PBS) for 45 min. Slides were then incubated with a polyclonal rabbit anti-E-cadherin or -NF-κB antibody (1:50, Cell Signaling, Danvers, MA, USA) for 2 h, washed with PBS and incubated with an Alexa 546-labeled goat anti-rabbit antibody (1:250, Molecular Probe, Eugene, Oregon, CA, USA) for 45 min. After rinsing in PBS, Hoechst 33258 (Molecular Probe) was added to slides for 15 min to counterstain nuclei. Slides were washed with PBS and mounted with Vectashield (Vector laboratories, Inc., Burlingame, CA, USA), and then visualized using a fluorescence microscope (Carl Zeiss, Oberkochen, Germany).

### Transient transfectin of nuclear factor-kappa B (NF-κB) cDNA

Transfection of NF-kB cDNA was performed using a Lipofectamine 2000 reagent (Invitrogen, Carlsbad, CA, USA) according to the manufacturer’s instructions. Plasmid (pcDNA-FLAG-Nv-NF-kB-Cys) was procured from Addgene (Cambridge, MA, USA). After incubation for 24h, the medium was removed, and the cells were washed with PBS followed by treatment with either gas (He+O_2_) only, 1 kV of NTP, 10 μg/ml of cetuximab, and NTP (1 kV for 1 s) plus cetuximab (10 μg/ml).

### Statistical analyses

One-way analysis of variance (ANOVA) following a post hoc Tukey’s test was performed using the SPSS 20.0 statistical software (SPSS, Chicago, IL, USA). Parameters of the data from three independent experiments are expressed as the means ± S.D. *P* < 0.05 was considered to indicate statistical significance (*P < 0.05; **P < 0.01; ***P < 0.001).

## Additional Information

**How to cite this article**: Chang, J. W. *et al.* Combination of NTP with cetuximab inhibited invasion/migration of cetuximab-resistant OSCC cells: Involvement of NF-κB signaling. *Sci. Rep.* **5**, 18208; doi: 10.1038/srep18208 (2015).

## Supplementary Material

Supplementary Dataset 1
