# Supplementary material for: Combination of NTP with cetuximab inhibited invasion/migration of cetuximab-resistant OSCC cells: Involvement of NF-κB signaling
Source: Sci Rep. 2015 Dec 14;5:18208. doi: 10.1038/srep18208 (PMC4677387; doi:10.1038/srep18208)
Supplement: Supplementary Dataset 1 [file srep18208-s1.doc]

**Supplementary Information**

**Combination of NTP with cetuximab inhibited invasion/migration of cetuximab-resistant OSCC cells: Involvement of NF-κB signaling**

Jae Won Chang^a^, Sung Un Kang^b^, Yoo Seob Shin^b^, Seong Jin Seo^b^, Yeon Soo Kim^b^, Sang Sik Yang^c^, Jong-Soo Lee^d^, Eunpyo Moon^d^, Keunho Lee^e^, and Chul-Ho Kim^b^

^a^Department of Otolaryngology-Head and Neck Surgery, College of Medicine, Chungnam National University, Daejeon, Republic of Korea

^b^Department of Otolaryngology, School of Medicine, ^c^Department of Electrical and Computer Engineering, ^d^Department of Molecular Science and Technology and Department of Life Science, Ajou University, Suwon, Republic of Korea

^e^PSM America Inc., Colorado Springs, CO, USA

Correspondence and requests for materials should be addressed to C.H.K.

(ostium@ajou.ac.kr)

**Supplementary Figure S1**


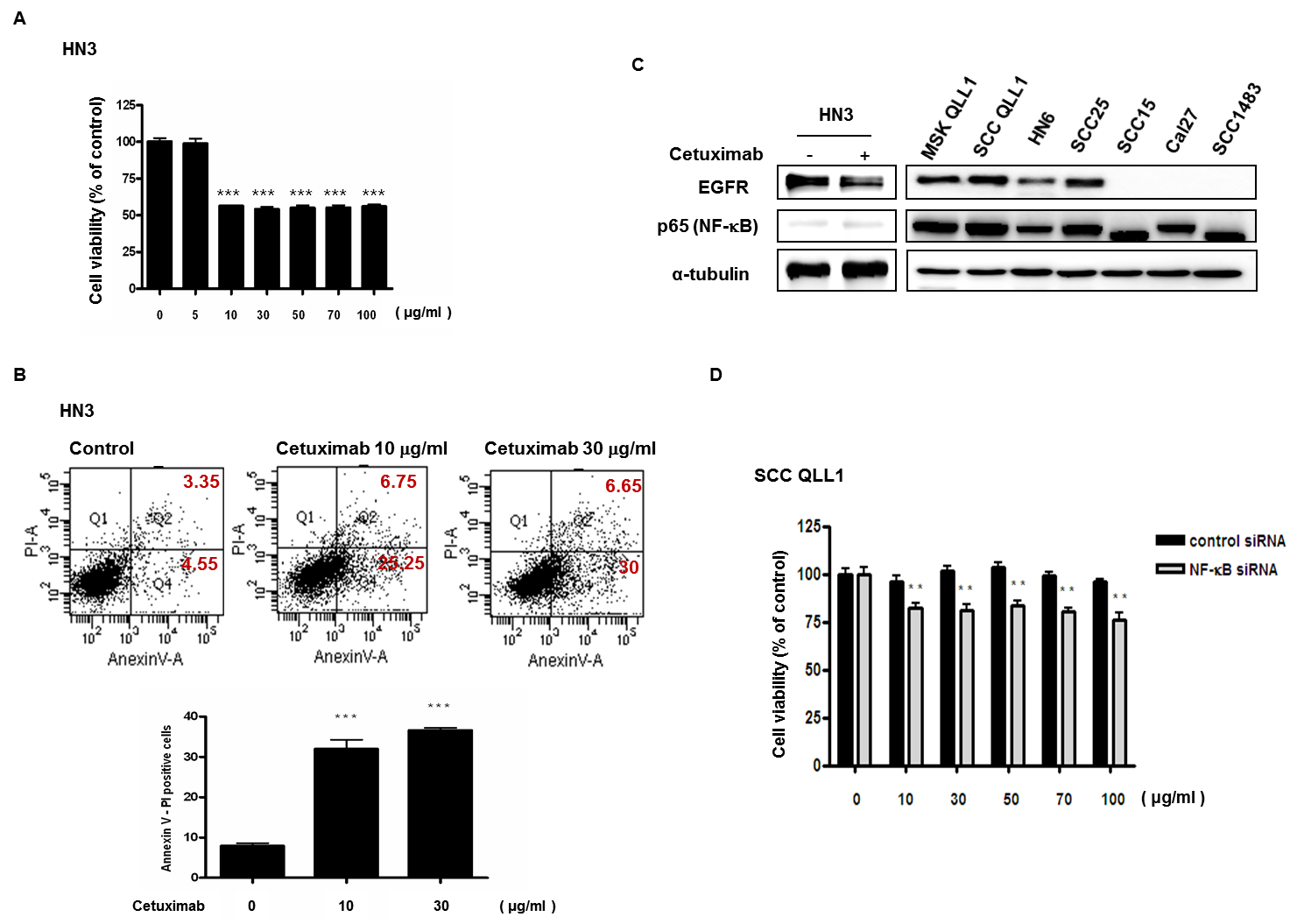


**Supplementary Fig. S1. NF-kB overexpression associated with cetuximab resistance.** (A) Cell viability assay (MTT) and (B) Annexin V/PI FACS analysis suggested that HN3 cells are sensitive for cetuximab (more than 10 μg/ml) treatment. (C) p65 (NF-kB) expression status on various cell lines. HN3 cells which is sensitive for cetuximab treatment showed scarce NF-kB expression whereas cetuximab resistance cells showed NF-kB overexpression on western blotting analysis. (D) Down regulation of NF-kB using RNA interference recovered cetuximab sensitivity on cetuximab-resistance SCCQLL1 cells. The data is representative of three experiments performed in triplicate.

**Supplementary Figure S2**

**
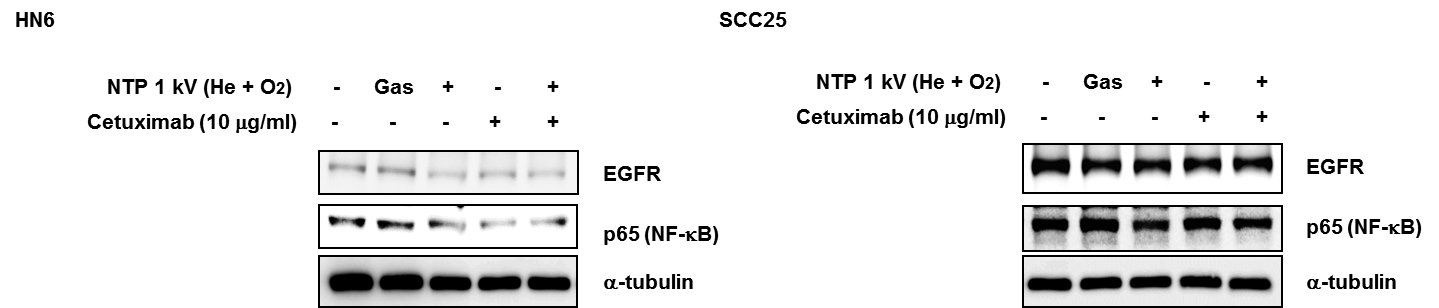
**

**Supplementary Fig. S2. Combination effect of NTP with cetuximab in other EGFR-expressing OSCC cell lines.** Combination of NTP with cetuximab decreased p65 (NF-kB) expression in HN6 cells whereas no definite p65 expression change was noted in SCC25 cells. Each Western-blotting band is representative of three experiments performed in triplicate.

**Supplementary Figure S3**


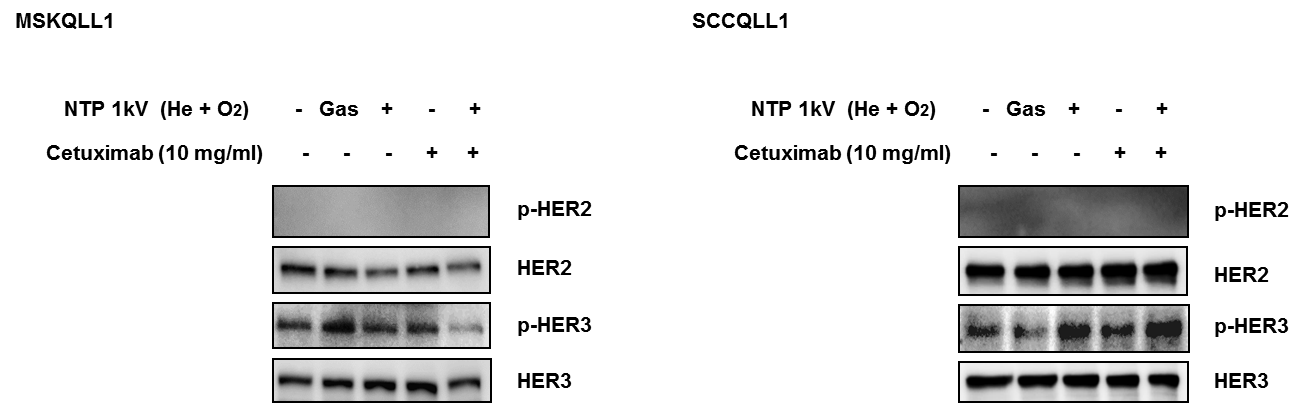


**Supplementary Figure S3. Combination effect of NTP with cetuximab appears to be linked to other mechanism such as HER-3 axis rather than NF-kB in MSKQLL1 cells.** In MSKQLL1 cells, the phosphorylation levels of HER3 were significantly decreased in the NTP (1 kV) combination with cetuximab (10 μg/ml) treatment group compared with those in the control, gas, NTP or cetuximab-only groups, but not in SCCQLL1 cells. We can deduce from these data that in contrast with SCCQLL1 cells, in MSKQLL1 cells, other mechanisms such as the HER-3 pathway appear to be linked to the effect of treatment with the combination of NTP with cetuximab. Each Western-blotting band is representative of three experiments performed in triplicate.
